# Promoter methylation analysis of O6-methylguanine-DNA methyltransferase in glioblastoma: detection by locked nucleic acid based quantitative PCR using an imprinted gene (*SNURF*) as a reference

**DOI:** 10.1186/1471-2407-10-48

**Published:** 2010-02-18

**Authors:** Luca Morandi, Enrico Franceschi, Dario de Biase, Gianluca Marucci, Alicia Tosoni, Mario Ermani, Annalisa Pession, Giovanni Tallini, Alba Brandes

**Affiliations:** 1 Department of Haemathology and Oncological Sciences Section of Pathology, Bellaria Hospital, University of Bologna, Italy; 2 Medical Oncology and Radiotherapy Departments, Bellaria-Maggiore Hospital, Azienda Unità Sanitaria Locale of Bologna, Italy; 3 Neurosciences Department, Statistic and Informatic Unit, Azienda Ospedale-Universita' of Padova, Italy; 4 Department of Experimental Pathology, University of Bologna, Italy

## Abstract

**Background:**

Epigenetic silencing of the *MGMT *gene by promoter methylation is associated with loss of *MGMT *expression, diminished DNA-repair activity and longer overall survival in patients with glioblastoma who, in addition to radiotherapy, received alkylating chemotherapy with carmustine or temozolomide. We describe and validate a rapid methylation sensitive quantitative PCR assay (MS-qLNAPCR) using Locked Nucleic Acid (LNA) modified primers and an imprinted gene as a reference.

**Methods:**

An analysis was made of a database of 159 GBM patients followed between April 2004 and October 2008. After bisulfite treatment, methylated and unmethylated CpGs were recognized by LNA primers and molecular beacon probes. The *SNURF *promoter of an imprinted gene mapped on 15q12, was used as a reference. This approach was used because imprinted genes have a balanced copy number of methylated and unmethylated alleles, and this feature allows an easy and a precise normalization.

**Results:**

Concordance between already described nested MS-PCR and MS-qLNAPCR was found in 158 of 159 samples (99.4%). The MS-qLNAPCR assay showed a PCR efficiency of 102% and a sensitivity of 0.01% for LNA modified primers, while unmodified primers revealed lower efficiency (69%) and lower sensitivity (0.1%). *MGMT *promoter was found to be methylated using MS-qLNAPCR in 70 patients (44.02%), and completely unmethylated in 89 samples (55.97%). Median overall survival was of 24 months, being 20 months and 36 months, in patients with *MGMT *unmethylated and methylated, respectively. Considering *MGMT *methylation data provided by MS-qLNAPCR as a binary variable, overall survival was different between patients with GBM samples harboring *MGMT *promoter unmethylated and other patients with any percentage of *MGMT *methylation (p = 0.003). This difference was retained using other cut off values for *MGMT *methylation rate (i.e. 10% and 20% of methylated allele), while the difference was lost when 50% of *MGMT *methylated allele was used as cut-off.

**Conclusions:**

We report and clinically validate an accurate, robust, and cost effective MS-qLNAPCR protocol for the detection and quantification of methylated *MGMT *alleles in GBM samples. Using MS-qLNAPCR we demonstrate that even low levels of *MGMT *promoter methylation have to be taken into account to predict response to temozolomide-chemotherapy.

## Background

Transcriptional inactivation by cytosine methylation at promoter CpG islands of tumour suppressor genes is thought to be an important mechanism in human carcinogenesis. A number of tumour suppressor genes, including *CDKN2A, MGMT, MLH1*, etc, are silenced by promoter methylation in a variety of tumors [1]. In the course of tumor development, gene silencing by DNA methylation is an early and important mechanism by which tumor-suppressor genes are inactivated [2,3].

Epigenetic silencing of the *MGMT *gene by promoter methylation is associated with loss of *MGMT *expression [4-6], diminished DNA-repair activity and longer overall survival in patients with glioblastoma (GBM) who, in addition to radiotherapy, received alkylating chemotherapy with temozolomide [7]. The *MGMT *gene is located on chromosome 10q26 and encodes a DNA-repair protein that removes alkyl groups from the O^6^position of guanine, an important site of DNA alkylation. The restoration of the DNA consumes the MGMT protein, which the cell must replenish. Left unrepaired, chemotherapy-induced lesions, especially O6-methylguanine, trigger cytotoxicity and apoptosis [8,9]. High levels of *MGMT *activity in cancer cells create a resistant phenotype by blunting the therapeutic effect of alkylating agents and may be an important determinant of treatment failure [3,8-13]. Patients with glioblastoma containing a methylated *MGMT *promoter showed a major benefit from temozolomide [14].

Given the key roles of cytosine methylation, there has been a wide interest in the development of procedures for DNA methylation analyses [2,3,6,15-26].

Vlassenbroeck I et al. [27] described a Real Time by SYBRGreen method to detect *MGMT *methylation status. The copy number of the methylated *MGMT *promoter, normalized to the *ACTB *gene, provides a quantitative test result. Woidacz TK et al. showed that *MGMT *methylation could be detected at levels as low as 0.1%. by high resolution melting analysis [28].

Here we present a novel methylation sensitive quantitative real time PCR assay (MS-qLNAPCR) which permits high throughput quantification of the methylation status of the *MGMT *promoter in an accurate, very sensitive and cost-effective manner. High specificity was achieved recognizing methylated and unmethylated CpGs by 3'-locked nucleic acid (LNA) primers and molecular beacon probes [29]. The CpG islands of *SNURF *were selected as a reference locus. *SNURF *belongs to the 15q imprinted center mapped on 15q12. The maternal allele is usually methylated, while the paternal one is unmethylated [30]. In theory in a homogeneous population of cells of the same individual the methylated maternal alleles should be balanced with the unmethylated paternal alleles if the tumor cells did not acquire any deletion for this locus or aberrant methylation of the paternal allele. This feature allows an easy and precise calculation of the ratio between the methylated and unmethylated alleles of *MGMT *following the method described by Ginzinger et al. [31].

## Methods

### Tumor Samples

For this study, samples were retrieved from the Pathology Section of the University of Bologna at Bellaria Hospital (Bologna). Tumors were classified and graded according to 2007 WHO [32] criteria. The use of brain tumor tissue after completing histopathological diagnosis for research purposes was approved by the regional ethics committee.

Following patient charts review, a retrospective analysis was made of a database of GBM patients followed prospectively between April 2004 and October 2008. We evaluated only patients who met the following inclusion criteria: age ≥ 18 years; Performance Status (PS) at diagnosis, 0-2; histological diagnosis of newly diagnosed GBM; *MGMT *methylation status assessed by methylation specific nested PCR; postoperative treatment consisting of radiotherapy (RT) followed by adjuvant temozolomide (TMZ) (14 patients) or TMZ concurrent with and adjuvant to RT (145 patients) [7]. Twenty-four blood samples from healthy donors were used as controls.

### DNA isolation

Tissue blocks were selected for DNA extraction after careful examination on hematoxylin and eosin staining of corresponding sections to exclude contaminating necrotic debris. Molecular genetic analyses were performed on samples showing an estimated tumor cell content of at least 90% from five sections of 10 μm from paraffin embedded tissue (FFPE) blocks. Tumor area was macrodissected manually by a sterile blade or were microdissected using the laser assisted SL μcut Microtest (MMI GmbH, Glattbrugg, Switzerland) as previously described[33]. Two incubations with xylene at 60°C and two incubations with absolute ethanol at room temperature for ten minutes each were used to eliminate paraffin. The tissues were then incubated with NaSCN 1 M for one hour at 37°C and lysed with proteinase K at 55°C overnight. Genomic DNA was extracted using the GENTRA Puregene tissue kit (Qiagen, Hilden, Germany) in accordance to the manufacturer's instructions. The pellet was then eluted in 35 μl of TE buffer and total DNA was quantified by Quant-iT™ dsDNA BR kit (Invitrogen, Carlsbad, California). At least 200 ng of DNA was then treated with bisulfite using the EpiTect Bisulfite kit (Qiagen, Hilden, Germany) according to the manufacturer's instructions.

### *In Vitro *Methylation Assay and Standards

To test for sensitivity and specificity of MS-qLNAPCR, titration experiments were performed using normal pooled genomic DNA (DNA Female pool, Cod. G1521, Promega, Madison, Wisconsin) which was methylated *in vitro *using *SssI *(New England Biolabs, Ipswich, MA). In brief, 1.5 μg was treated with *Sss*I to methylate all CpG sites (near complete methylation and no loss of DNA was assumed) for two hours at 37°C following the instruction of the provider. Mixtures of *Sss*I-treated DNA: untreated DNA (100%, 50%, 10%, 1%, 0.1% 0.01%) were prepared in duplicate (each containing 1.5 μg of template DNA). 50% *Sss*I-treated DNA: untreated DNA served as calibrator for *MGMT *MSP-qPCR, while the same DNA amount of untreated DNA pool (Promega, Madison, Wisconsin) served as calibrator for *SNURF *MSP-qPCR to confirm equal amounts of maternal methylated allele and paternal unmethylated allele. The same DNA pool was used to test for specificity in terms of absence of amplicons for the *MGMT *methylated allele for single runs.

### MS-nested PCR and MS-qLNAPCR

Nested MS-PCR was performed as previously described [34,35] with minor modifications: a total of 26 cycles for the flanking primers and a total of 30 cycles for the methylation specific primers were performed. Amplicons were detected by SeaKem LE agarose gel (3%, Lonza, Basel, Switzerland) by the use of GelStar (Lonza, Basel, Switzerland) as intercalator. Real Time PCR analysis was performed using an SDS-ABI Prism 7000 (Applied Biosystems, Foster City, CA). 3'-locked nucleic acid (LNA) primers (see Table 1) were synthesized by SIGMA-Proligo (SIGMA-Proligo, Paris, France). Reactions were performed in a final volume of 25 μl, adjusted to 4 mM of MgCl_2 _and containing 1 Unit of FastStartTaq DNA Polymerase (Roche, Mannheim, Germany), 1× related Buffer and 5 μl of 5× GC rich solution, 200 μM of dNTPs, 0.5 μl of ROX 50× (Invitrogen, Carlsbad, California), 500 nM of each primers, 250 nM of beacon probe and 3 μl of bisulfite treated DNA. The real time qPCR cycling conditions were as follows: 95°C for 4 min, 60°C for 2 min, 72°C for 2 min, followed by 40 cycles for 20 s at 95°C, 45 s at 60°C with fluorescence measurement, 30 s at 72°C.

**Table 1:** primers and beacon probes used in this study:

gene	Primer forward	Beacon probe	Primer reverse
MGMT METHYLATED	5'-TTT**C**GA**C**GTT**C**GTAGGTTTT**C**G+**C-**3'	5'-FAM- CCGGAGCGTAT**C**GTTTG**C**GATTTGGTGAGTGTGCTCCGG-BHQ1-3'	**5'-G**CACTCTTCC**G**AAAAC**G**AAAC+**G-**3'

MGMT UNMETHYLATED	5'-TTTGTGTTT**T**GA**T**GTT**T**GTAGGTTTT**T**G+**T-**3'	5'-FAM-CCGGTGCTGTAT**T**GTTTG**T**GATTTGGTGAGTGTGCACCGG-BHQ1-3'	5'-AACTCC**A**CACTCTTCC**A**AAAAC**A**AAAC+**A-**3'

SNURF METHYLATED	5'-GGATTTTTGTATTG+**C**GGTAAATAAGTA+**C-**3'	5'-FAM- CCGGAGGGAGGTAGGTTGG**C**G**C**GTATGTTTAGCCTCCGG-BH1-3'	5'-C**G**CTACAACAAC+**G**ACAAACTTC+**G-**3'

SNURF UNMETHYLATED	5'-GGATTTTTGTATTG+**T**GGTAAATAAGTA+**T-**3'	5'-FAM- CCGGAGGGAGGTAGGTTGG**T**G**T**GTATGTTTAGGCCTCCGG-BH1-3'	5'-CTC**A**CTACAACAAC+**A**ACAAACTTC+**A-**3'

SNURF FLANKING PRIMERS	GGGAGTT+GGGATTTTT+GTATTG		CTCCC+CAAACTATCT+CTTAAAAAAAA

(LNA nucleotides are preceeded by +; CpG discriminatory LNA nucleotides are in bold)

### Relative Quantification of the Methylated Allele

The number of PCR cycles (*Ct*) required for the FAM intensities to exceed a threshold just above background was calculated for the test (*MGMT*) and reference (*SNURF*) reactions as described previously [31] with some modifications: *Ct *values were determined for each sample and subtracted to obtain:

(m*MGMT*: methylated allele; u*MGMT*: unmethylated allele; m*SNURF*: methylated allele; u*SNURF*: unmethylated allele).

ΔCt values were measured for each unknown sample [ΔCt (test DNA)] and for calibrator [ΔCt (equal amount of *Sss*I-treated DNA mixed with the same amount of untreated DNA)].

Relative copy number at each locus in the test sample was then calculated as:

Relative Methylated allele copy number = (1+*E*)^-ΔΔCt ^where:

ΔΔCt = ΔCt (test DNA) - ΔCt (calibrator DNA), and *E *= PCR efficiency. The efficiency of PCR was calculated from the slope of the line, E = 10^-1/slope ^- 1. We used primers with PCR efficiencies of >95% and We calculated the relative DNA copy number ratio respect the unmethylated allele following the formula = 2^-*ΔΔCt *^as previously described [31].

A sample of 24 healthy controls was used to estimate the normality range of the differences between methylated and unmethylated *SNURF *alleles; the normality range was estimated as the mean value ± 1.96 * standard deviation.

Results were considered invalid if criteria of DNA quantity and quality were not met. These were based on minimal starting amplifiable DNA of approximately 6.25 ng (equivalent of ~1000 copies of a diploid human genome) available for amplification after bisulfite treatment and calculated by ABI 7000 SDS software using the standard curve for u*MGMT*. For this purpose, serial dilutions of normal female DNA (DNA Female pool, Cod. G1521, Promega, Milan) were tested by u*MGMT *primers to create a standard curve and calculate the starting amount of each DNA specimen. Positive signals from u*MGMT*, m*SNURF *and u*SNURF *were considered as internal controls for PCR reaction in order to exclude the presence of PCR inhibitors. Cases below a threshold of approximately 6.25 ng were repeated starting from a higher amount of tumor tissue. Specimens showing a threshold below this limit were considered invalid and were excluded from the study. The investigators who performed the MS-qLNAPCR tests were blinded to the clinical data and patient outcome.

### Primers and molecular beacon probes design

**Figure 1:**
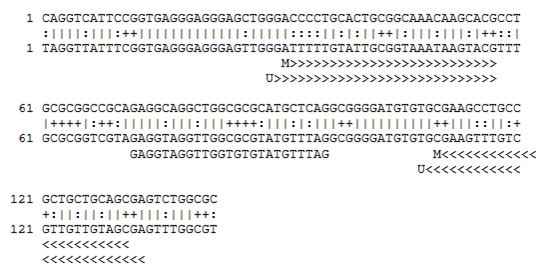
**CpG island of *SNURF *promoter**. The upper lane identifies the exact sequence before bisulfite modification. The bottom lane represents the bisulfite treated methylated forward sequence where all C's are changed to T's, except for those followed by a G. Primer sequences are underlined by >. The internal probe covers two consecutive CpGs (marked as +).

Previously described primers for methylated and unmethylated *MGMT *promoter [14,19,35] were modified only to insert LNA nucleotides at the 3' end of their sequence (See Table 1). Primers for *SNURF *were designed using MethPrimer http://www.urogene.org/methprimer/index1.html[36] and verified by methBlast http://medgen.ugent.be/methBLAST (see Fig. 1 and Table 1).

Molecular beacon probes (see Table 1) were designed using the Primer3 software http://frodo.wi.mit.edu/cgi-bin/primer3/primer3_www.cgi including two CpG sites each. Flanked molecular beacon arms were designed using the OLIGO 6.0 software reaching a temperature between 57°C and 61°C in the stem loop conformation. LNA nucleotide were added to primers as described previously [37] using a *T*m prediction tool available at exiqon web site http://www.exiqon.com. Amplicons were tested by *MFOLD *http://www.bioinfo.rpi.edu/applications/mfold/old/dna/ in order to avoid secondary structures within primer and probe positions.

### Direct Sequencing

**Figure 2:**
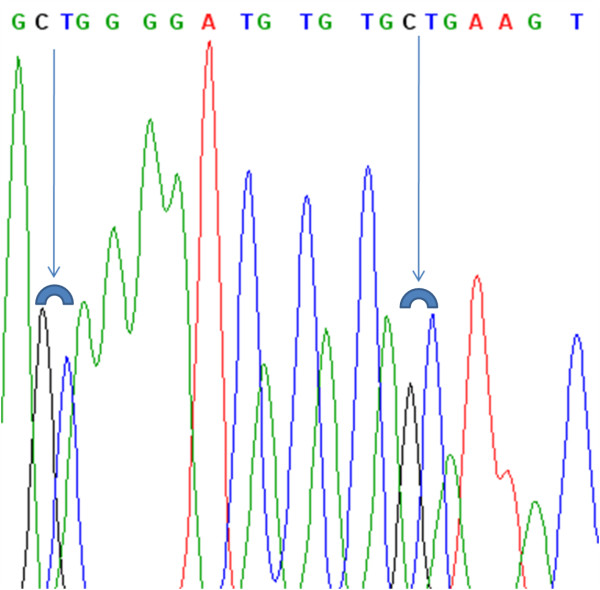
**Direct Sequencing of *SNURF *promoter**. Direct sequencing after bisulfite modification of two CpGs of *SNURF *for case BF215. The arrows indicates the simultaneous presence of C/T (C: black peak; T: blue peak) due to a balanced amount of methylated and unmethylated cytosine in the CpGs amplified with our primer set.

PCR primers to amplify the CpG island of *SNURF *promoter were designed by MethPrimer software on line http://www.urogene.org/methprimer/, which was also used to predict CpG islands and CpG sites in the sequence (see Table 1). The amplification of bisulfited-modified DNA was performed using FastStartTaq™ polymerase (Roche, Milan), with the following conditions: 95°C for 4 min, followed by 40 three steps cycles at 95°C for 30 sec, 60°C for 30 sec and at 72°C for 30 sec. The PCR products were separated on 3% SeaKem^® ^LE agarose gels and purified (Agencourt, Beverly, MA), followed by sequencing by CEQ2000XL automatic DNA sequencer (Beckman Coulter, Fullerton, CA). The presence of a cytosine residue after bisulfite treatment shows that the cytosine residue was protected by methylation from bisulfite modification (Fig. 2).

## Results

159 patients (M:F 99:60, median age: 57 years, range 25-77 years) met the inclusion criteria. TMZ concurrent with and adjuvant to RT was administered in 145 patients (91.2%). *MGMT *promoter was found methylated by nested MS-PCR in 69 samples (43.39%), and unmethylated in 90 samples (56.60%), and was found methylated by MS-qLNAPCR in 70 patients (44.02%), and unmethylated in 89 samples (55.97%). Concordance between the two assays was found in 158 of 159 samples (99.4%). The only patient discordant was found unmethylated by nested MS-PCR and showed methylation in less than 1% of cells by MS-qLNAPCR (ratio between m*MGMT*/u*MGMT *= 0.0008).

**Figure 3:**
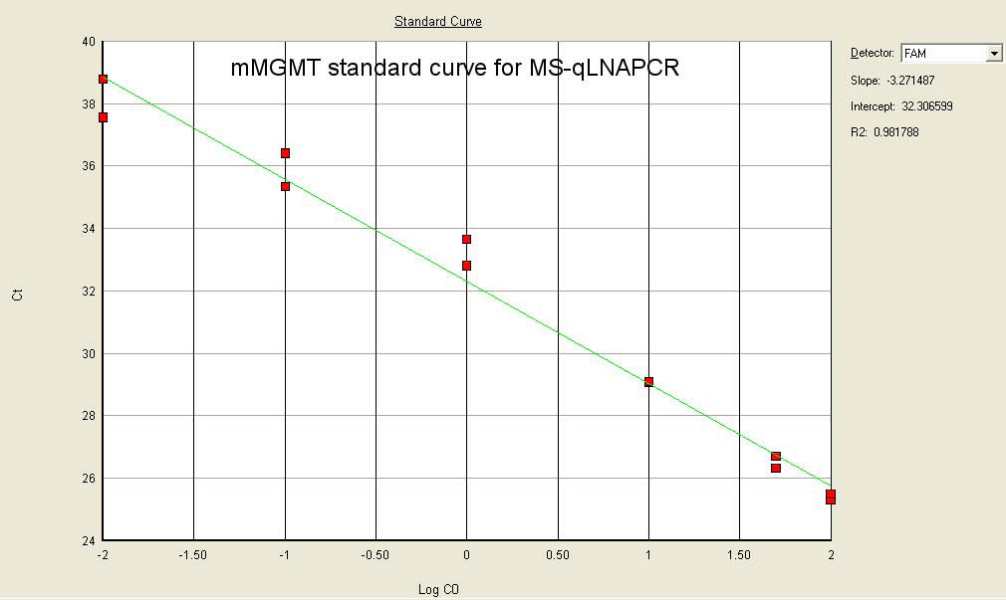
**Standard curves for MS-qLNAPCR with LNA primers**. MS-qLNAPCR standard curves with serial dilution mixtures of *Sss*I-treated DNA:untreated DNA (100%, 50%, 10%, 1%, 0.1%, 0.01%, 0.001%; each containing 1.5 μg of template DNA) for LNA primers specific for m*MGMT*. LNA modified primers for m*MGMT *show higher PCR efficiency (slope: -3.271; efficiency: 102%) than conventional primers (slope: -4.339; efficiency: 69%, see Fig.4). The analytical detection limit of 0.01% is reached only with LNA primers.

**Figure 4:**
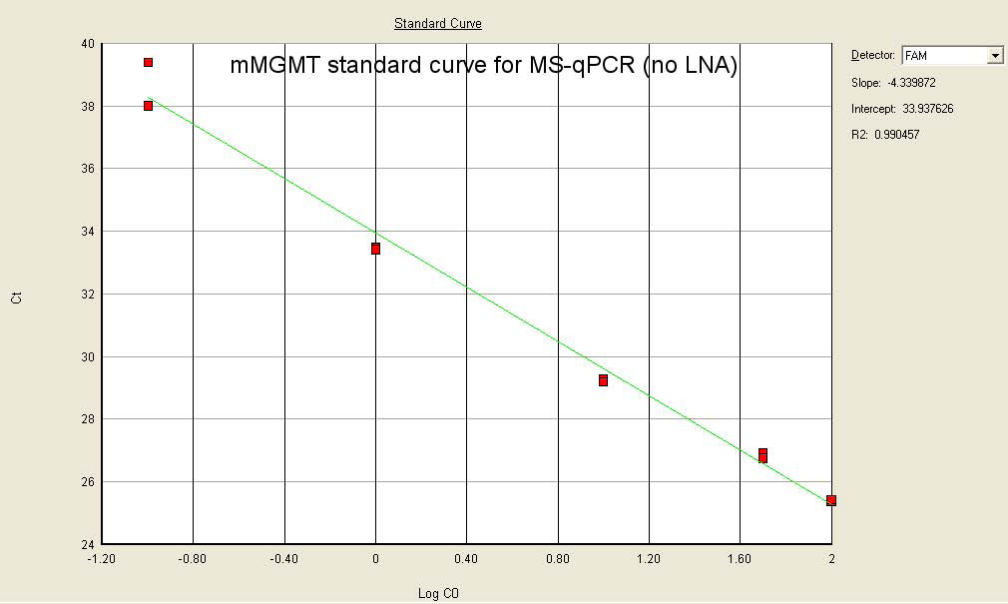
**Standard curves for MS-qPCR with conventional primers**. MS-qPCR standard curves with serial dilution mixtures of *Sss*I-treated DNA:untreated DNA (100%, 50%, 10%, 1%, 0.1%, 0.01%, 0.001%; each containing 1.5 μg of template DNA) for conventional primers[35] specific for m*MGMT*. Conventional primers for m*MGMT *show lower PCR efficiency (slope: -4.339; efficiency: 69%) than LNA modified primers (see Fig.3). The analytical detection limit with the use of conventional primers was 0.1%.

We tested in parallel two sets of primers specific for m*MGMT *and u*MGMT *with and without LNA modifications for PCR efficiency in the MS-qLNAPCR assay. LNA modified primers for m*MGMT *showed higher PCR efficiency (slope: -3.271; efficiency: 102%, see Fig. 3) than unmodified primers (slope: -4.339; efficiency: 69% see Fig. 4). The analytical detection limit of 0.01% was reached only for LNA modified primers, while conventional primers showed a detection limit of 0.1%. The analytical detection limit (sensitivity limit) of the MS-qLNAPCR assay was determined using the ABI7000™ instrument using serial dilution mixtures of *Sss*I-treated DNA: untreated DNA (100%, 50%, 10%, 1%, 0.1% 0.01% each containing 1.5 μg of template DNA).

1.5 μg of SssI treated DNA (hypothetically fully methylated DNA) maintained a small amount of unmethylated DNA for *MGMT *promoter (0.00079%). By this protocol a sensitivity of 0.01% methylated alleles was detected and confirmed by the 24 replicates with a 95% cut-off value, according to common diagnostic practices [38].

The specificity of the assays was assessed by purification and analysis of a series of DNA from whole blood of 24 healthy donors without detecting any methylated allele for MS-qLNAPCR. On the contrary we detected two methylated cases by nested MS-PCR.

The total failure rate of unmethylated allele PCR was 0%. Inhibitors were not observed due to the high efficiency of DNA extraction and bisulfite treated DNA purification.

During the evaluation tests, 12 replicates of dilution mixture of 0.01% *Sss*I-treated DNA in a background of 1.5 μg of untreated DNA were evaluated with no false negative results.

Evaluating *SNURF *data from 24 healthy blood donors, we found a mean difference between methylated and unmethylated *SNURF *alleles of 0.001 (SD = 0.81); the normality range was therefore estimated as [-1.57, +1.59]. Values outside the *SNURF *normality range were detected in 14 out of 159 GBM (8.8%; see the Additional file 1 for details). In these cases we could not calculate the methylated/unmethylated *MGMT *allele ratio because the requirement for using *SNURF *as a reference is that methylated and unmethylated *SNURF *alleles are at a ratio of 1:1.

The *SNURF *promoter of the 14 cases with Ct ratio [(m*SNURF *- u*SNURF*)] outside the normality range was sequenced to verify whether they showed the simultaneous presence of both methylated and unmethylated cytosine in each of the CpGs interrogated by the primers and beacon probes. Every specimen showed the presence of both methylated and unmethylated cytosine in the CpGs (see Fig. 1 and Fig. 2), demonstrating that the Ct ratios outside normalcy are not due to primer mismatch and different PCR efficiency for the methylated and unmethylated alleles. Of the 14 GBMs with Ct ratio outside the normality range only five among methylated cases had *MGMT *methylation and the *ΔCt *of *SNURF *had some impact on the final calculation for the m*MGMT*/u*MGMT *ratio in only one of 70 cases (1.42%, sample BF215). The *SNURF *promoter of BF215 was sequenced showing both methylated and unmehtylated cytosines for each of the CpGs tested (see Fig. 2). This indicates that the BF215 ratio of Ct [(m*SNURF *- u*SNURF*)] is not due to primer/probe mismatch but may be the result of a partial loss of imprinting (LOI) of the maternal allele (see Additional file 1 for details).

**Figure 5:**
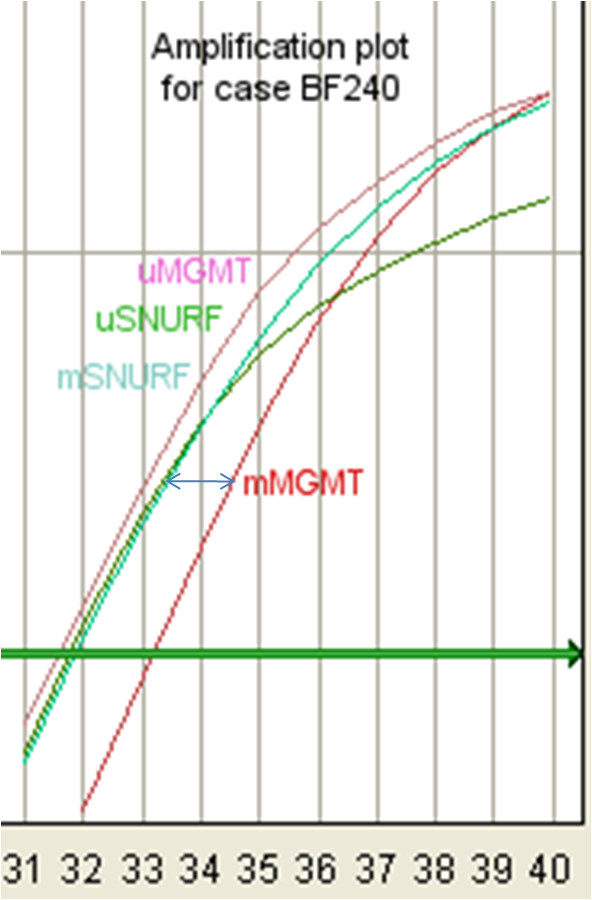
**A representative MS-qLNAPCR plot**. A representative MS-qLNAPCR plot (GBM case BF240) showing an *MGMT *methylated ratio value of 0.46.

**Figure 6:**
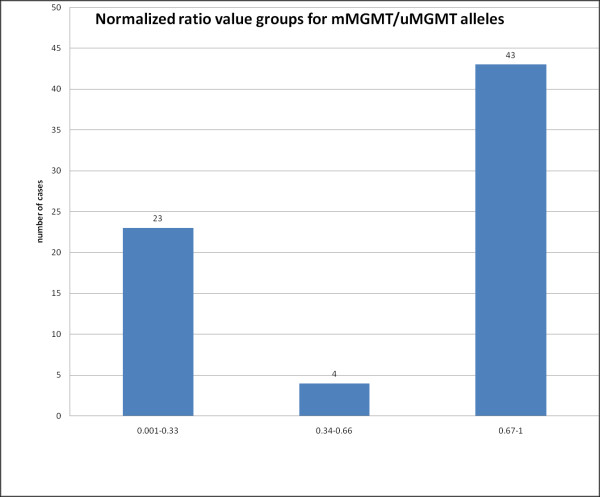
**Bimodal distribution of methylated and unmethylated alleles**. Normalized ratio value groups between methylated and unmethylated alleles of the 70 *MGMT *methylated GBMs identified with our MS-qLNAPCR protocol. There is a bimodal distribution of cases with two prevalent groups showing ratio values between 0.001-0.33 and 0.67-1, respectively.

With our MS-qLNAPCR protocol we identified 70 of 159 *MGMT *methylated cases (44.0%). In addition, MS-qLNAPCR allows a fine discrimination of the relative amounts of methylated vs. unmethylated *MGMT *in a given case (see Fig. 5). Quantitative analysis showed a bimodal distribution of ratio values between methylated and unmethylated *MGMT *alleles, with two prevalent groups with ratios between 0.001-0.33 and 0.67-1, respectively (see Fig. 6). This bimodal distribution is similar to that found by Vlassenbroeck et al. [27].

### Survival

**Figure 7:**
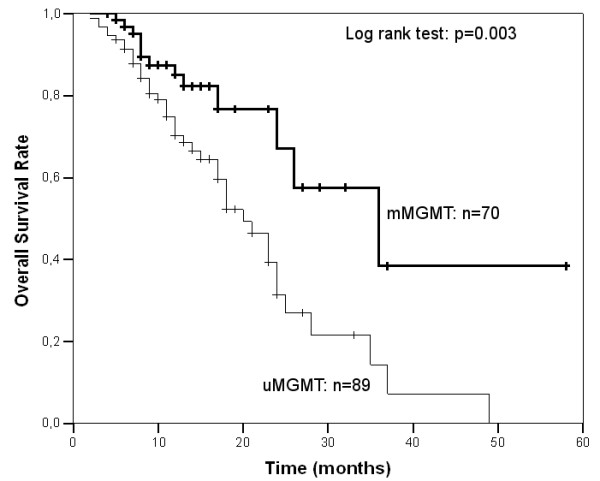
**Kaplan-Meier survival curves**. Survival curves of GBM patients with methylated *MGMT *promoter (thick line) and unmethylated *MGMT *promoter (thin line) determined by MS-qLNAPCR. Patients with tumors with unmethylated *MGMT *promoter have lower overall survival (Log rank test p = 0.003).

Median overall survival of the 159 patients was of 24 months (95%CI: 21 - 27), being 20 months and 36 (95%CI: 17.7 - 54.3) months, in patients with *MGMT *unmethylated and methylated tumors by nested MS-PCR, respectively (log-rank test, p = 0.004). Considering *MGMT *methylation data provided by our MS-qLNAPCR protocol as a binary variable, overall survival was statistically different between patients with GBM samples harboring *MGMT *promoter unmethylated and other patients with any percentage of *MGMT *methylation (log-rank test, p = 0.003, Fig. 7). This difference was retained using other cut off values for *MGMT *methylation rate (i.e. 10% and 20% of methylated cells), while the difference was lost when 50% of *MGMT *methylated cells was used as cut-off. Overall survival significantly correlated with *MGMT *promoter methylation status determined either by nested MS-PCR (p = 0.021) or MS-qLNAPCR (p = 0.025) even after the 14 patients that received RT followed by adjuvant TMZ were excluded.

## Discussion

Given the key roles of cytosine methylation, there has been a wide interest in the development of procedures for DNA methylation analyses [15]. The prognostic significance of *MGMT *promoter methylation has been shown in two chemoradiotherapy clinical trials; first in a phase II study testing concomitant and adjuvant temozolomide and radiation [39] and subsequently in EORTC 26981/22981 & NCIC CE.3 [14]. In the later study, *MGMT *promoter methylation was an independent favourable prognostic factor and patients whose tumour contained a methylated *MGMT *promoter had median survival of 21.7 months and 2-year survival of 46%, when treated with temozolomide and radiotherapy. These studies suggest that determination of *MGMT *methylation status maybe an important factor in determining which glioblastoma patients should receive chemoradiotherapy [40], but its prognostic significance in the routine clinical setting is not clearly established. Methylation-specific PCR (MSP) is widely used to test *MGMT *promoter methylation; however, in EORTC 26981/22981 & NCIC CE.3 Hegi et al. [14] only achieved methylation data for 67% of samples analysed, representing 36% of cases.

Sensitive, accurate, high-throughput and cost-effective methods should give a better definition of the role and control of this epigenetic modification, especially in cancer. Many different experimental approaches have been developed to allow either global large-scale or specific analyses [2,3,6,15-23]. The most popular approaches rely on bisulfite treatment of DNA [41]. This treatment is performed in such a way that, while cytosine is quantitatively deaminated to uracil, 5-methyl cytosine remains unmodified, thus allowing identification of the cytosine methylation status following PCR amplification. There are many possible variations in the subsequent DNA sequence detection methods used, which achieve diverse levels of performance and adequacy. The quantitative analysis of the cytosine methylation levels at several independent proximal positions can be achieved using Pyrosequencing analysis of PCR products amplified in a methylation-independent manner [18,41]. This procedure is not particularly sensitive and does not perform well with neoplastic populations that represents <10% of the sample, which limits its usefulness in the analysis of heterogeneous pathological samples and of early events in epigenetic reprogramming. MS-PCR [42] is the most widely used assay for the detection of hypermethylation in CpG islands. It relies on the selective PCR amplification of sequences corresponding to either unmethylated or methylated DNA using primers that anneal specifically with either one of the DNA species. For methylation-specific annealing, each primer must contain sequences corresponding to at least two CpG dinucleotides. This method allows sensitive detection of particular methylation patterns and is currently used for the analysis of pathological samples.

MS-PCR has been adapted to fluorescence-based real-time PCR technology, thus allowing for both quantitative and high-throughput sample analysis [17,18,27,28,42,43].

Eads CA et al. introduced quantitative methylation sensitive PCR based on TaqMan^® ^chemistry [24], determining the relative amounts of a particular methylation pattern with quantitative accuracy. Hattermann K et al. [44] described a method combining methylation specific and SYBR green based quantitative PCR allowing the quantification of fully methylated and fully unmethylated *MGMT *DNA species. Values were related to standard curves, corrected for DNA input by an internal calibrator, and calculated in relation to methylated and unmethylated control DNAs as a percentage share [44]. Smith E et al. [26] described an elegant and cost effective quantitative methylation sensitive PCR based on melting curve analysis obtained after amplification of bisulfite modified DNA in a real-time thermocycler. Vlassenbroeck I et al. [27] validated a direct quantitative methylation sensitive PCR assay based on SYBRGreen detection chemistry, which quantifies the copy number of the methylated *MGMT *promoter after normalization of Ct values with the *ACTB *reference gene. To the best of our knowledge, we are the first to describe and validate a method to quantitate DNA methylation using LNA modified primers and an imprinted gene as a reference, instead of a methylation independent calibrator such as *ACTB *[27]. In our opinion *ACTB *does not represent the best reference gene for normalization because it is located at 7p15-p12, a chromosomal site subject to copy number variations in gliomas [45,46], and because it is close *EGFR *(located at 7p12) that is amplified in about 40% of GBMs [47]. We use of an imprinted gene (*SNURF*) as an internal control, because it may check the efficiency of the assay from DNA purification, through bisulfite treatment to PCR. Additionally it should be used as a reference because *mSNURF *and *uSNURF *mimic the biallelic *MGMT *status. In fact, in normal cells the maternal allele of *SNURF *is methylated at the promoter locus, while the paternal allele of the same gene is usually unmethylated and expressed [30]. This condition is thus similar to a tumor population of cells in which the *MGMT *is methylated at one of the two alleles. In order to consider *SNURF *as an ideal reference, the ratio between methylated and unmethylated *SNURF *alleles might not be disturbed by copy number changes, or by loss of imprinting, both of which are common in cancer. However, several references demonstrate that *SNURF*, which has been mapped at 15q12, is hardly ever altered in gliomas [45,48-51]. These data were confirmed by our study because the methylated and unmethylated *SNURF ΔCt *of the most part of cases (91.2%) was nearly always very close to 0. The *SNURF *methylation values outside the normality range in 14 of the 159 GBMs (8.8%) may be due to a distinct CpG methylation pattern among tumor cell population, to partial loss of one allele (LOI: loss of imprinting), or to a methylation machinery disorder that methylates the paternal allele (GOI: gain of imprinting). The requirement for using *SNURF *as a reference is that methylated and unmethylated *SNURF *alleles are at a ratio of 1:1, and in our series in only one of these 14 cases did the *ΔCt *of *SNURF *have a negative impact on the final calculation for the m*MGMT*/u*MGMT *ratio. In these circumstances we avoid using *SNURF *as a reference loosing relative quantification data.

By sequencing the *SNURF *promoter of this abnormal case (BF215, Fig. 2), we found a balanced ratio between methylated and unmethylated cytosine in CpG interrogated loci. These data demonstrate for this case that the *ΔCt *found to be outside the normality range is not due to primer/probe mismatch but may be the result of a partial LOI of the maternal allele.

LNA based PCR has been widely used to achieve high specificity for the detection of single nucleotide polymorphism (SNP). LNA primers show very accurate mismatch discrimination in comparison with conventional DNA primers at all 3'-terminal positions [37]. Moreover, they offer greater sensitivity with respect to TaqMan based SNP detection due to the fact that in allele specific PCR for SNP detection two PCR amplify selectively each allele. On the other hand, in case of TaqMan technology a single PCR with flanking primers will amplify in parallel both alleles. If one allele is poorly represented (e.g. in samples with low density of tumor cells in a background of normal cells) the TaqMan probe may fail to detect or underestimate the less represented allele.

Because of these reasons we have taken advantage of 3'-LNA modified primers and molecular beacon detection chemistry [52,53] to developed a direct methylation sensitive quantitative PCR (MS-qLNAPCR) assay. Although allele specific assays provide an elegant method to discriminate between alleles, quantification of the variants is not attainable because of the intrinsic endpoint detection by conventional PCR. This limitation is fully addressed using real-time qPCR methods [54], whereby PCR product accumulation is monitored at each PCR cycle by means of fluorescent detection using molecular beacon chemistry. Unlike previously described methylation sensitive quantitative PCR protocols [27,28], our new MS-qLNAPCR approach is based on two different detection beacon probes, one recognizing the methylated allele and the other one recognizing the unmethylated allele.

These probes were designed to span two CpGs in order to obtain high specificity compared with previous MS-PCR or MS-qPCR methods with SYBRGreen detection, with high discriminatory power between the methylated and unmethylated *MGMT *alleles even with DNA extracted from FFPE samples. Variation of standard curves run in parallel with tumor samples in each batch of cases were small and acceptable, indicating optimal reproducibility. We found our MS-qLNAPCR protocol particularly reliable when dealing with large amounts of input DNA since it avoids false positive results [37].

To limit experimental variation we chose to utilize the same primers used by Hegi et al. which have been clinically validated on a large number of samples [14,19,35]. We modified the primers only to insert the LNA nucleotides to improve the assay and demonstrated that our LNA modified primers show optimal PCR efficiency and high sensitivity, better than that of large clinical studies [14]. These have so far largely used nested MS-PCR. MS-qLNAPCR offers many advantages compared with nested MS-PCR, including ease of interpretation compared with gel-based methods where weak bands are often difficult to interpreted, quantitative results, a one step procedure that saves time and reduces contamination risk, a broad dynamic range with reproducible results with both high and very low input DNA amounts. Low DNA amounts could still be processed also because the use of the EpiTect Bisulfite kit reduced the intrinsic DNA fragmentation during the bisulfite treatment. The advantages of our MS-qLNAPCR protocol make it thus ideal for high throughput analysis, when large numbers of clinical samples need to studied.

Vlassenbroeck I et al. [27] using MS-qPCR have shown that the amount of *MGMT *promoter methylation in GBMs is variable, with a bimodal distribution of cases. With MS-qLNAPCR we have fully confirmed their data. In addition we demonstrate that epigenetic silencing of *MGMT *is associated with response to temozolomide-chemotherapy even when the amount of the methylated *MGMT *allele is low. This is very important for the clinical implications of a molecular diagnosis of *MGMT *promoter methylation in GBMs. Since low levels of *MGMT *promoter methylation retain a predictive value, even weak methylation signals should be reported. The biological explanation of the phenomenon is unclear, but it should be kept in mind that, next to *MGMT*, other factors may be involved in temozolomide response (e.g. the high activity of poly-ADP ribose polymerase and base excision repair machinery [55,56]).

## Conclusions

We report and validate clinically, a novel, accurate, robust, and cost effective MS-qLNAPCR protocol for the detection and quantification of methylated *MGMT *alleles. This protocol is the only quantitative method validated to date from both technical and clinical standpoints for GBM samples. Using MS-qLNAPCR we demonstrate that even low levels of *MGMT *promoter methylation have to be taken into account to predict response to temozolomide-chemotherapy.

## Abbreviations

CDKN2A: cyclin-dependent kinase inhibitor 2A; MGMT: *O6*-Methylguanine-DNA methyltransferase; MLH1: mutL homolog 1; GBM: Glioblastoma; LNA: locked nucleic acid; RT: Radiotherapy; TMZ: temozolomide; FFPE: paraffin embedded and formalin fixed; MS-PCR: methylation sensitive PCR; MS-qLNAPCR: methylation sensitive quantitative locked nucleic acid real time PCR; AS-PCR: allelic specific PCR; LOI: loss of imprinting; GOI: gain of imprinting.

## Competing interests

The authors declare that they have no competing interests.

## Authors' contributions

Conception and Design: LM; Provision of study materials or patients: AB, EF, AT; Collection and assembly of data: LM, DB, GM, AP, GT; Data analysis and interpretation: LM, EF, AT, AP, GT, ME, GM; Manuscript writing: LM, GT. Final approval of manuscript: LM, EF, GM, DB, AT, AP, GT, AB, ME

## Pre-publication history

The pre-publication history for this paper can be accessed here:

http://www.biomedcentral.com/1471-2407/10/48/prepub

## Supplementary Material

Additional file 1 **MS-qLNAPCR and nested PCR data comparison**. this file includes MS-qLNAPCR and nested PCR data for each of the 159 cases. GBM = glioblastoma; Nested-PCR: methylation status obtained by nested PCR; MS-qLNAPCR: methylation status obtained by MS-qLNAPCR. Met/Unmet Ratio: relative DNA copy number ratio between methylated and UnMethylated allele calculated from the formula described in Materials and methods; Ct [(mSNURF - uSNURF)]: delta Ct between SNURF methylated allele and SNURF UnMethylated allele. Click here for file
